# Supplementary figures and images for: A quantitative assessment of site-level factors in influencing Chukar (Alectoris chukar) introduction outcomes
Source: PeerJ. 2021 Apr 16;9:e11280. doi: 10.7717/peerj.11280 (PMC8054752; doi:10.7717/peerj.11280)

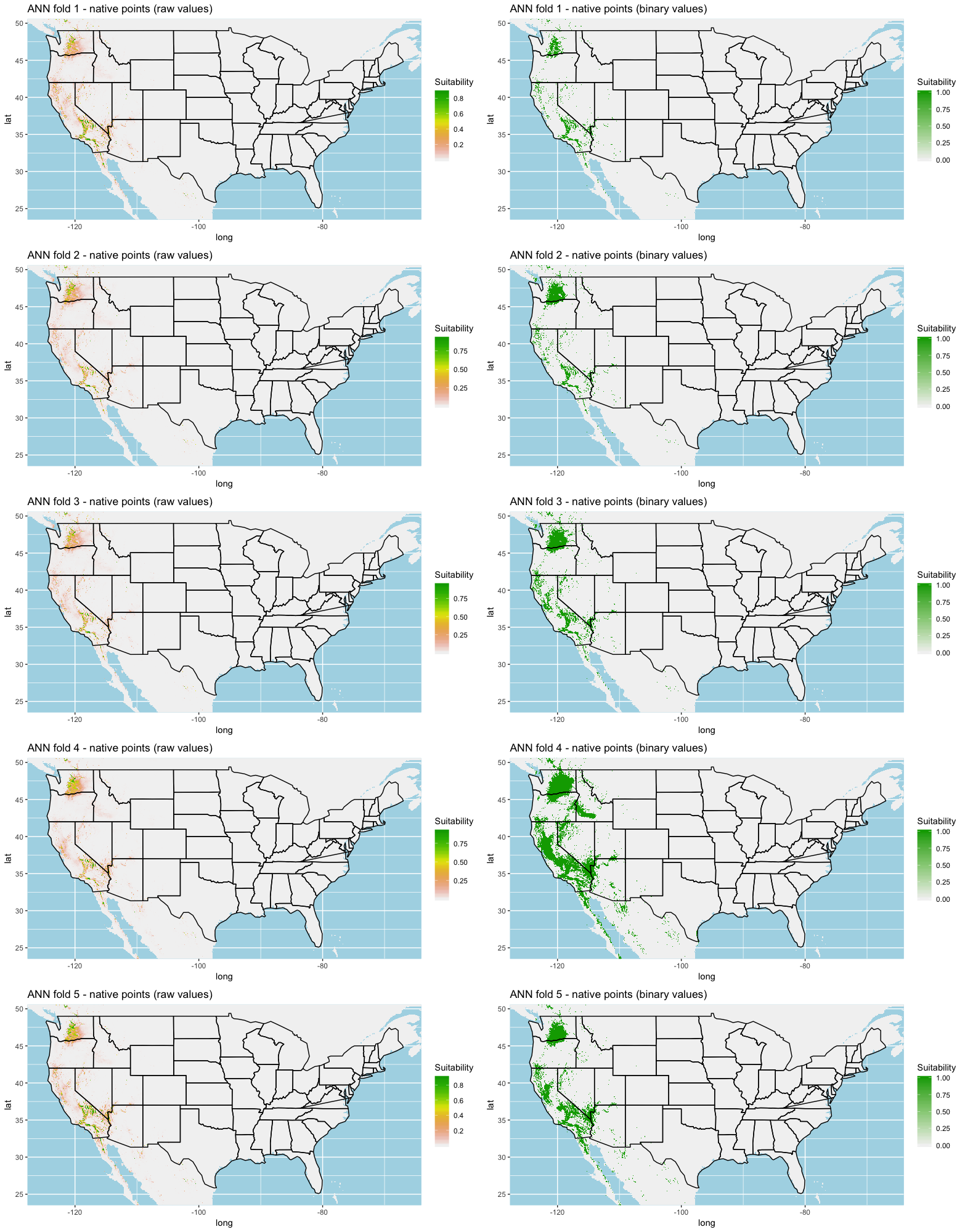

Supplement: Supplemental Information 1 [file peerj-09-11280-s001.png]

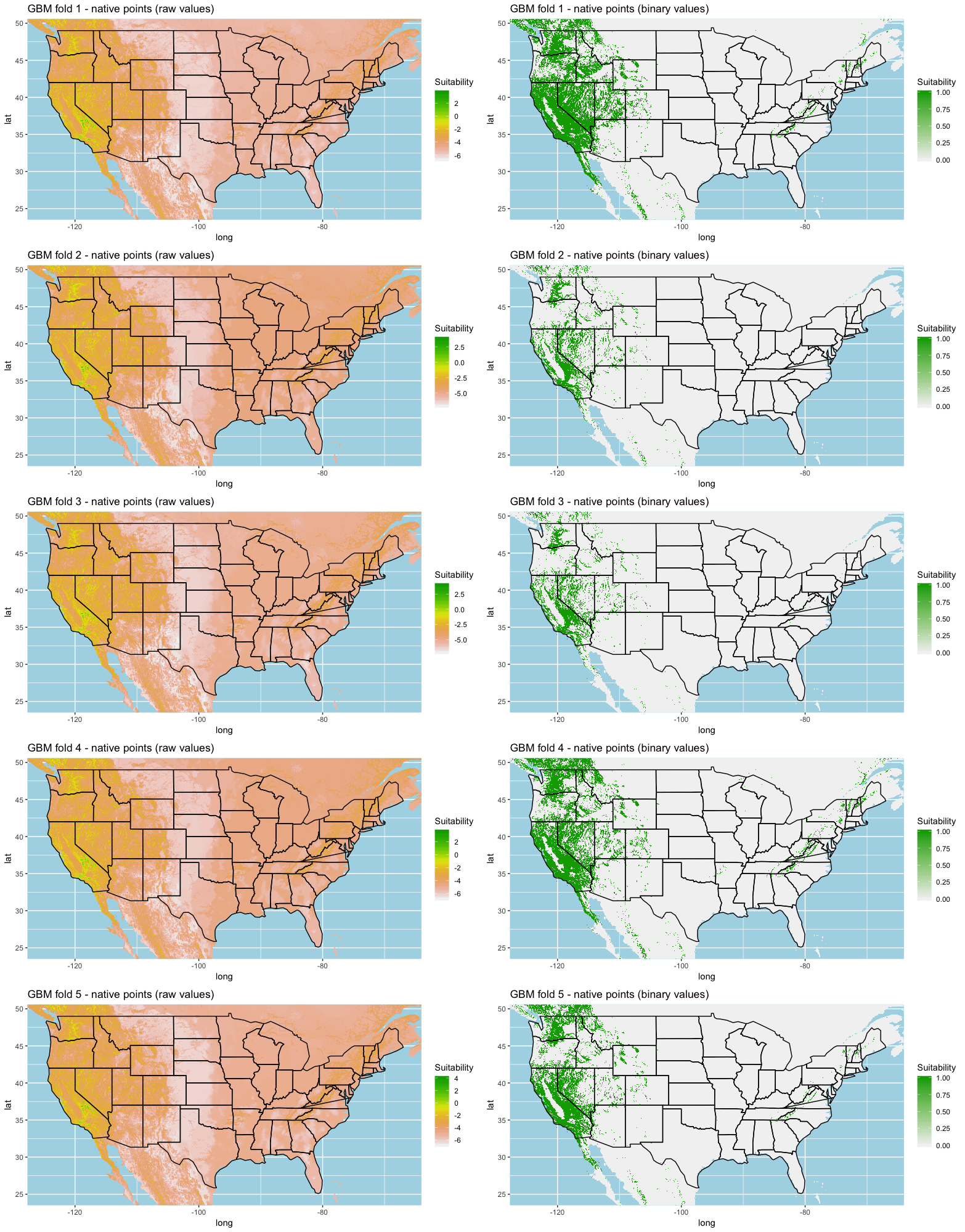

Supplement: Supplemental Information 2 [file peerj-09-11280-s002.png]

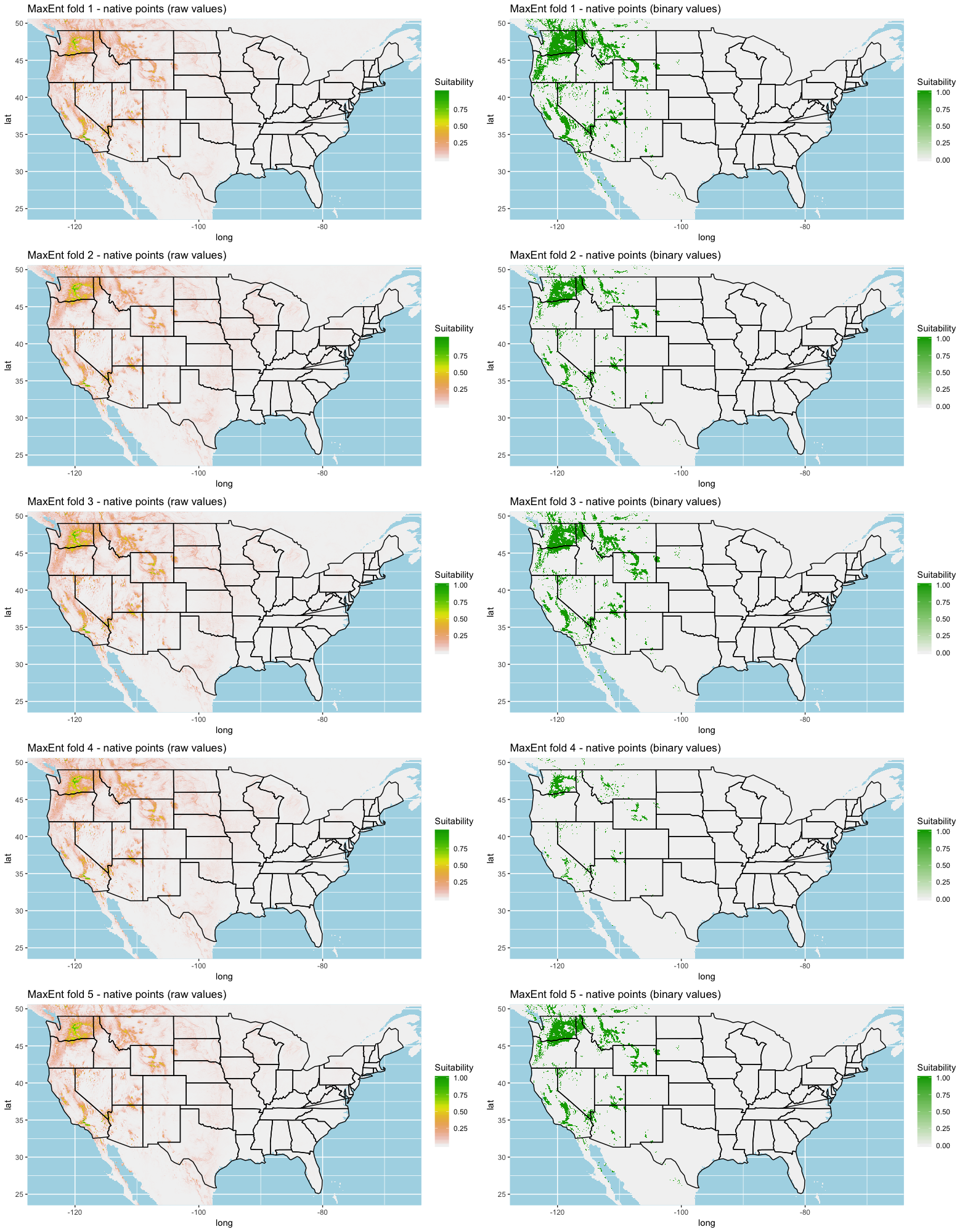

Supplement: Supplemental Information 3 [file peerj-09-11280-s003.png]

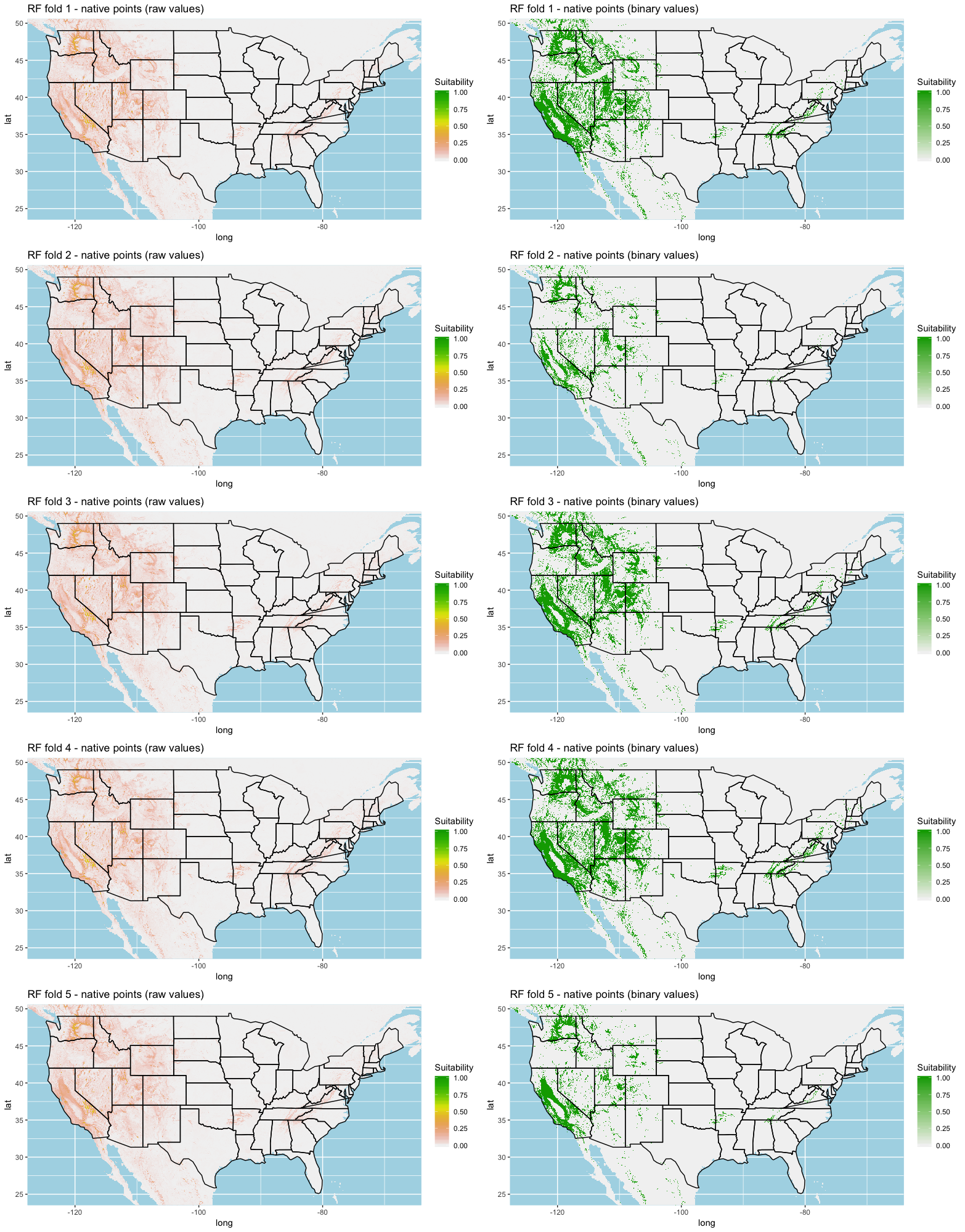

Supplement: Supplemental Information 4 [file peerj-09-11280-s004.png]

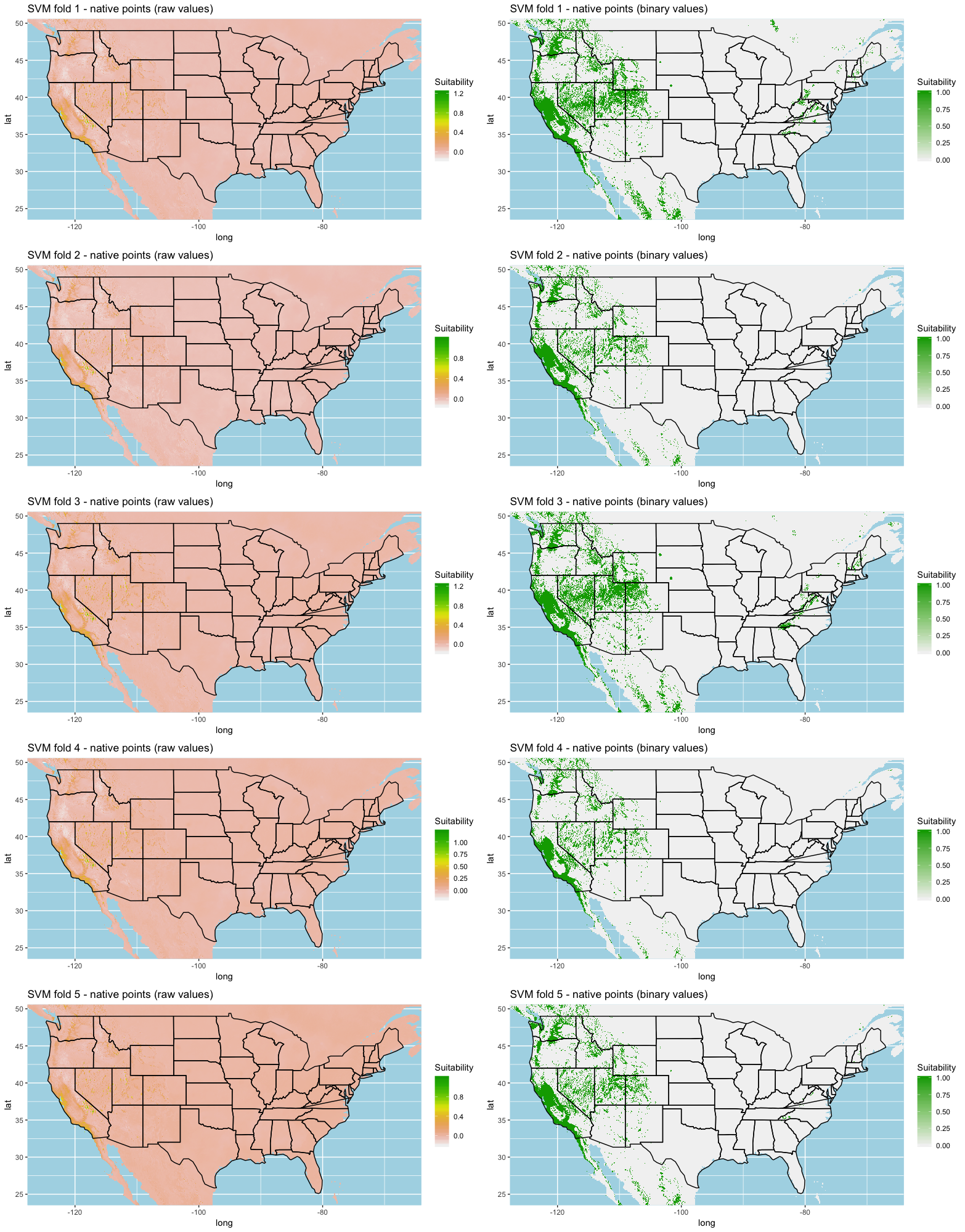

Supplement: Supplemental Information 5 [file peerj-09-11280-s005.png]

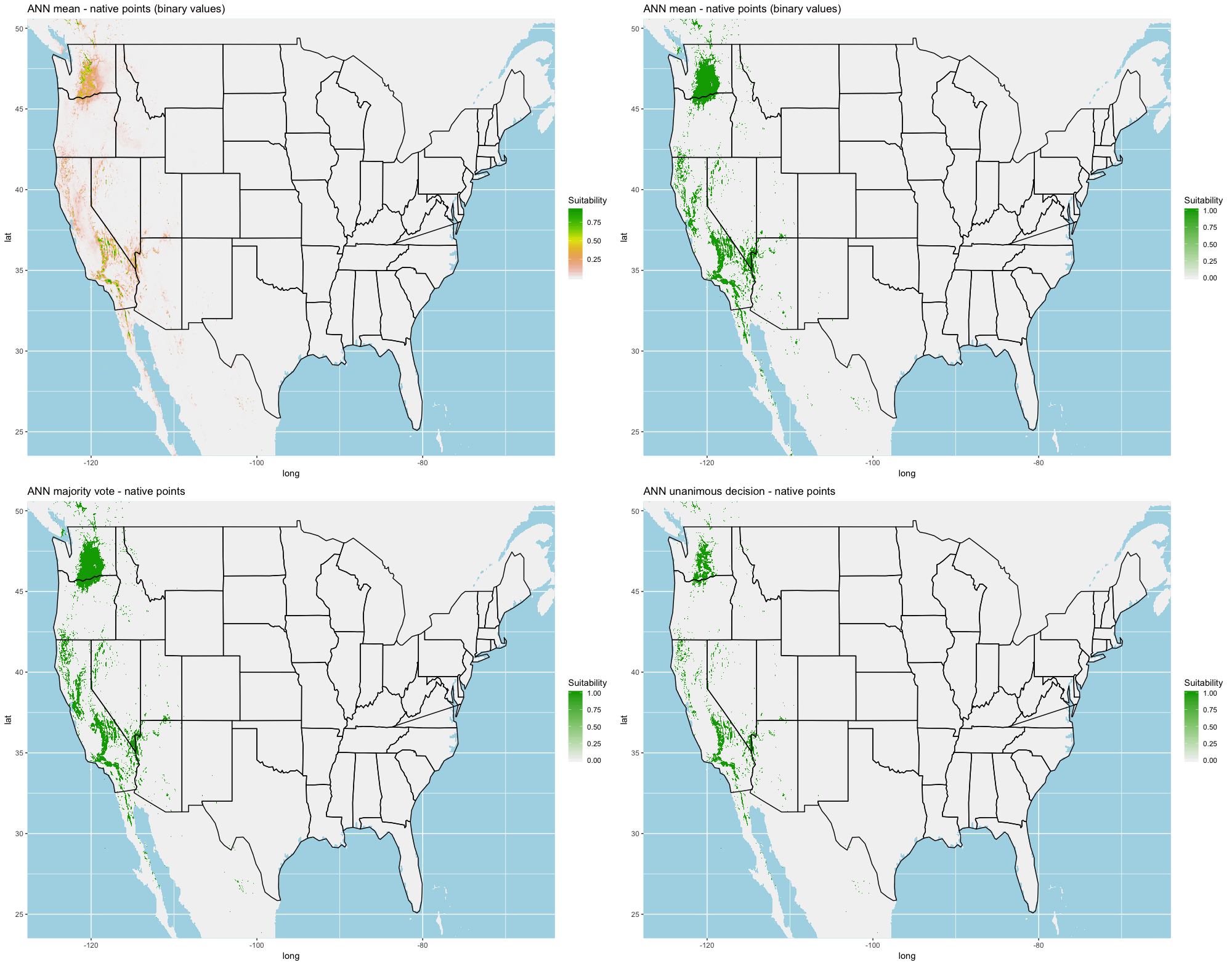

Supplement: Supplemental Information 6 [file peerj-09-11280-s006.png]

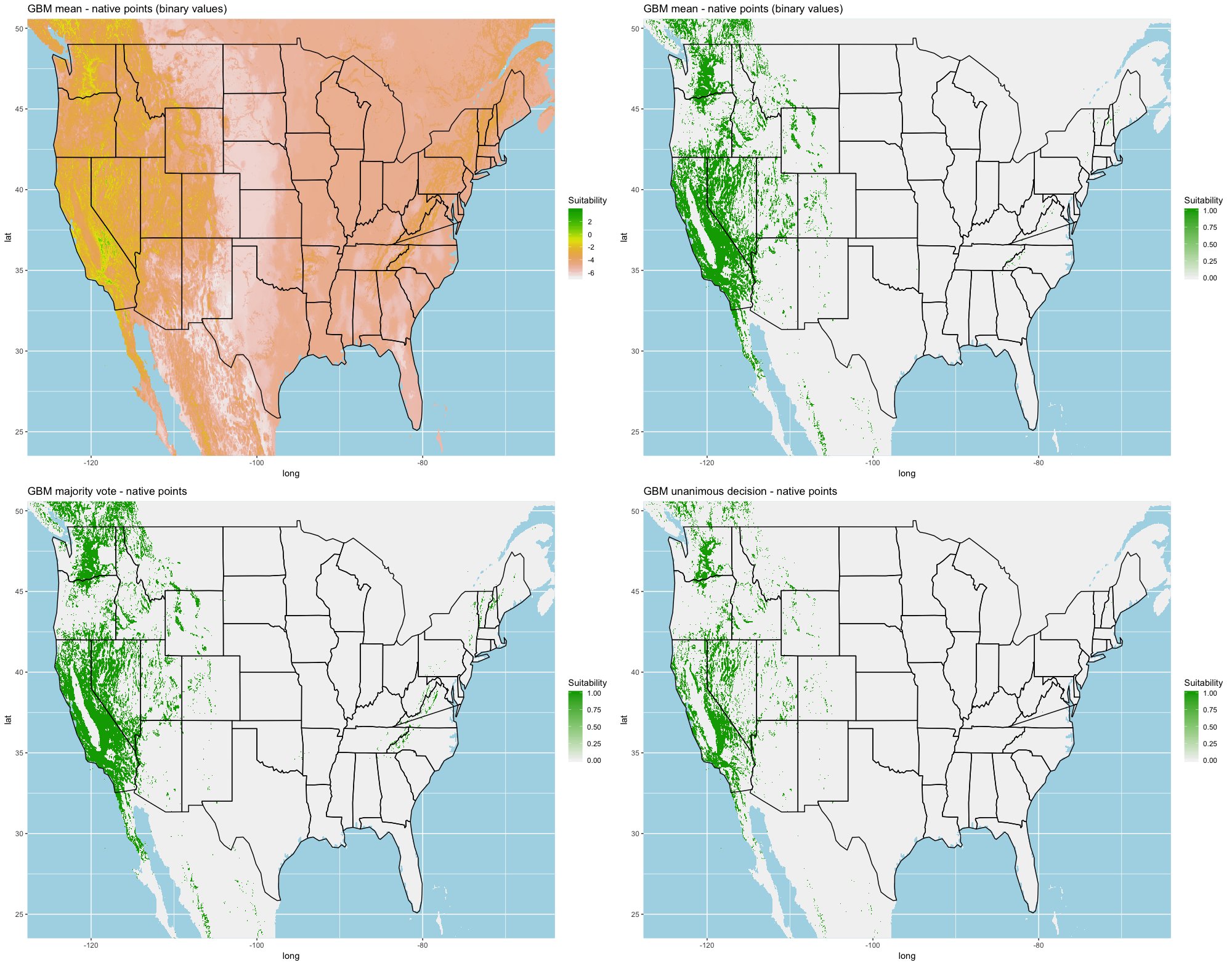

Supplement: Supplemental Information 7 [file peerj-09-11280-s007.png]

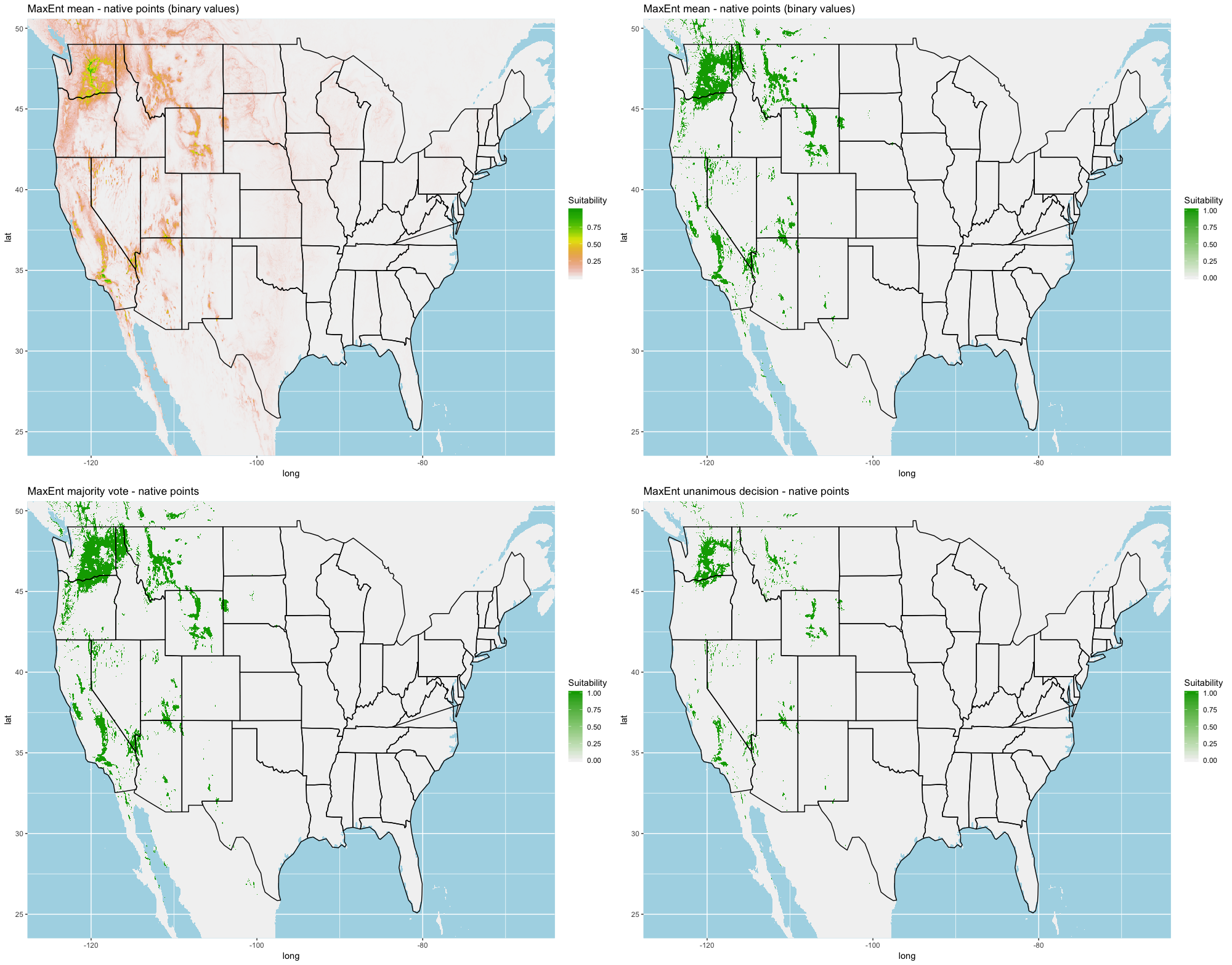

Supplement: Supplemental Information 8 [file peerj-09-11280-s008.png]

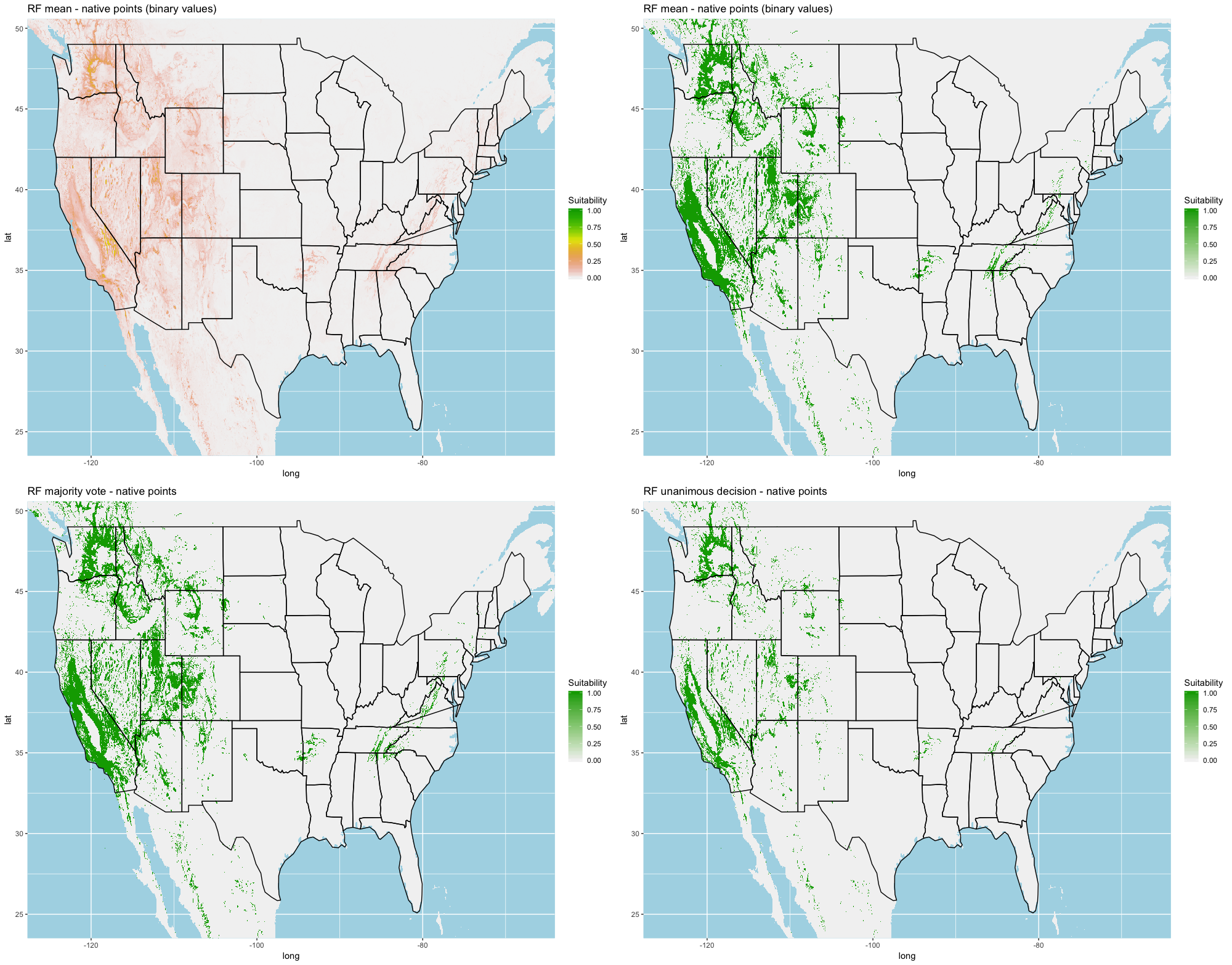

Supplement: Supplemental Information 9 [file peerj-09-11280-s009.png]

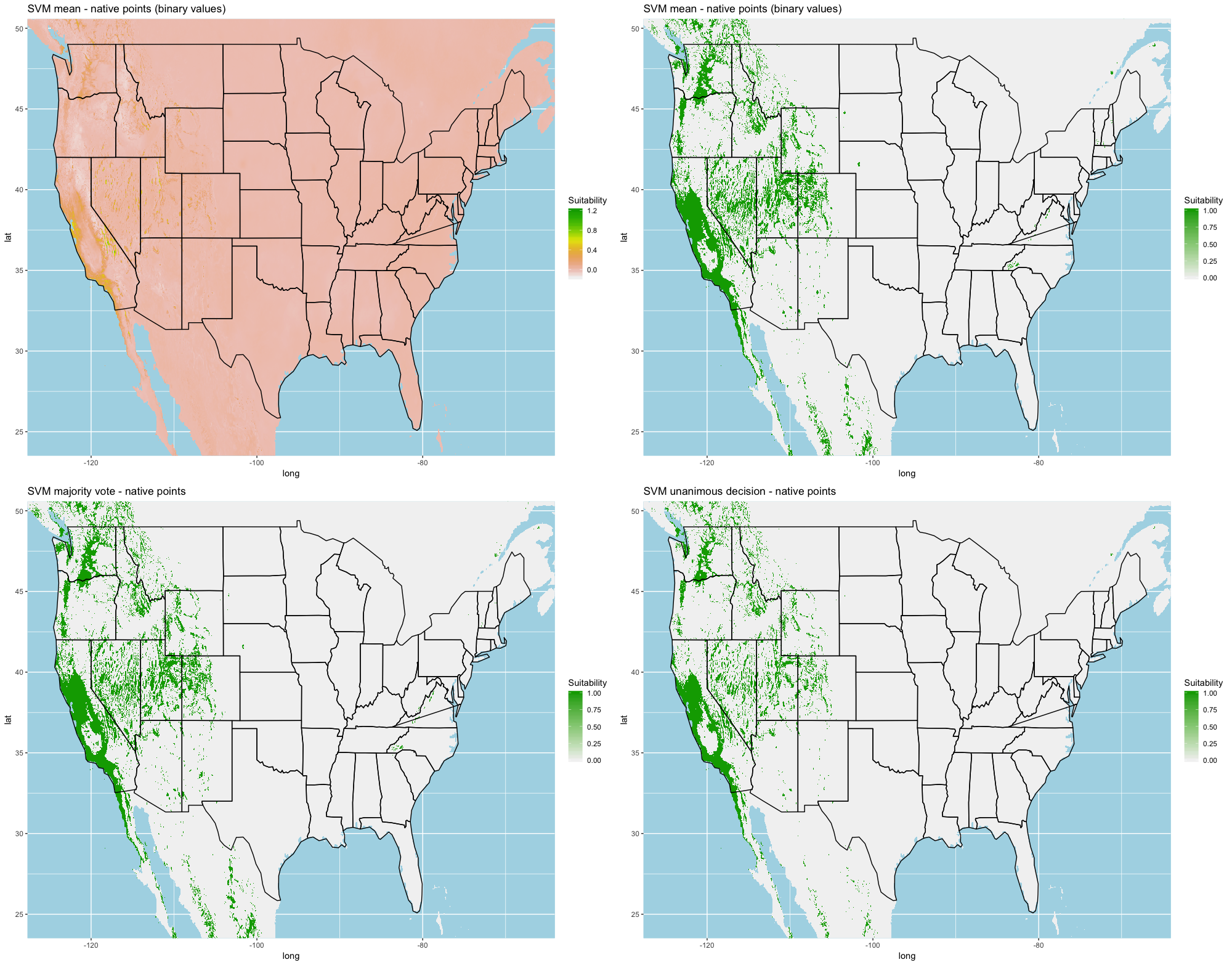

Supplement: Supplemental Information 10 [file peerj-09-11280-s010.png]

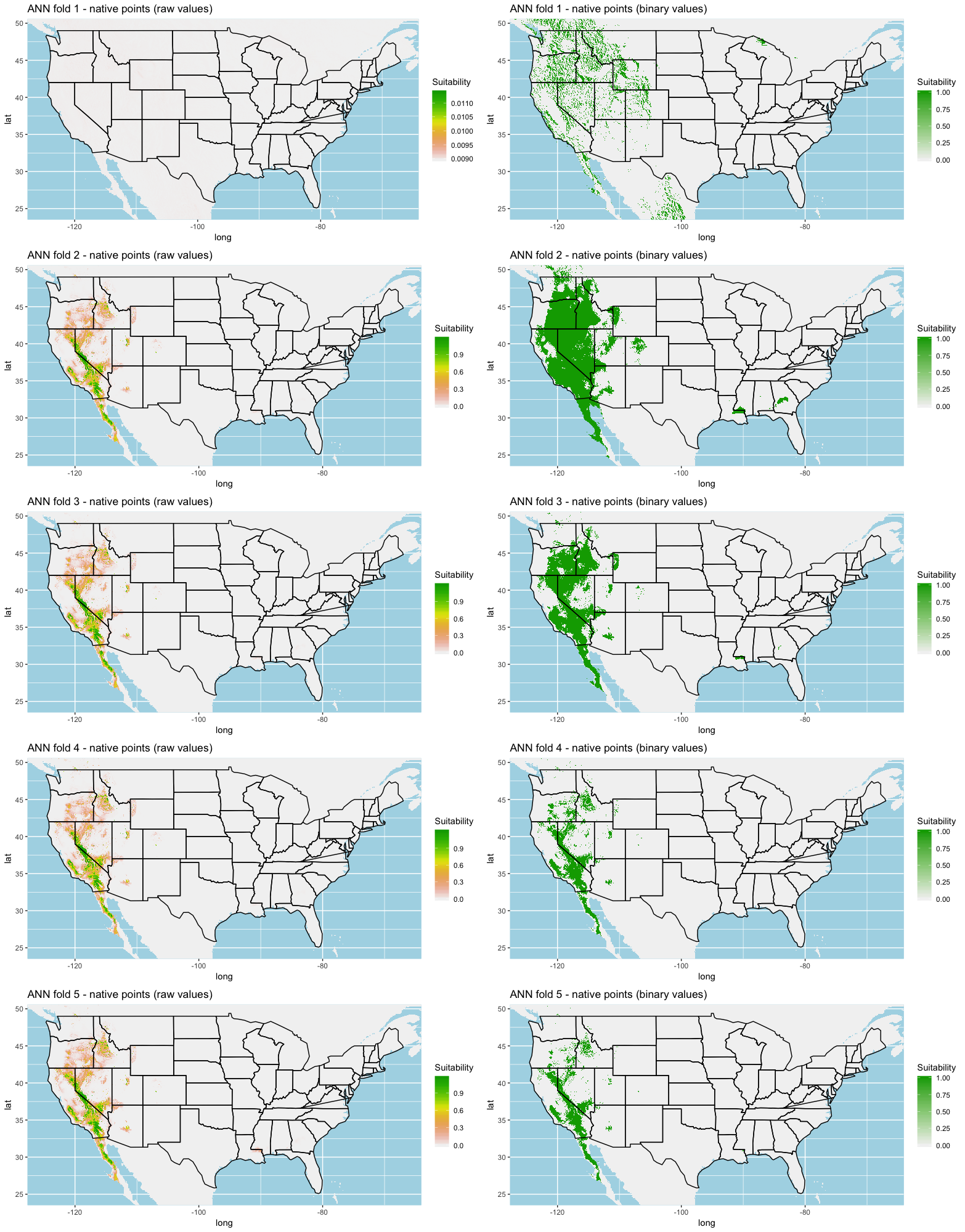

Supplement: Supplemental Information 11 [file peerj-09-11280-s011.png]

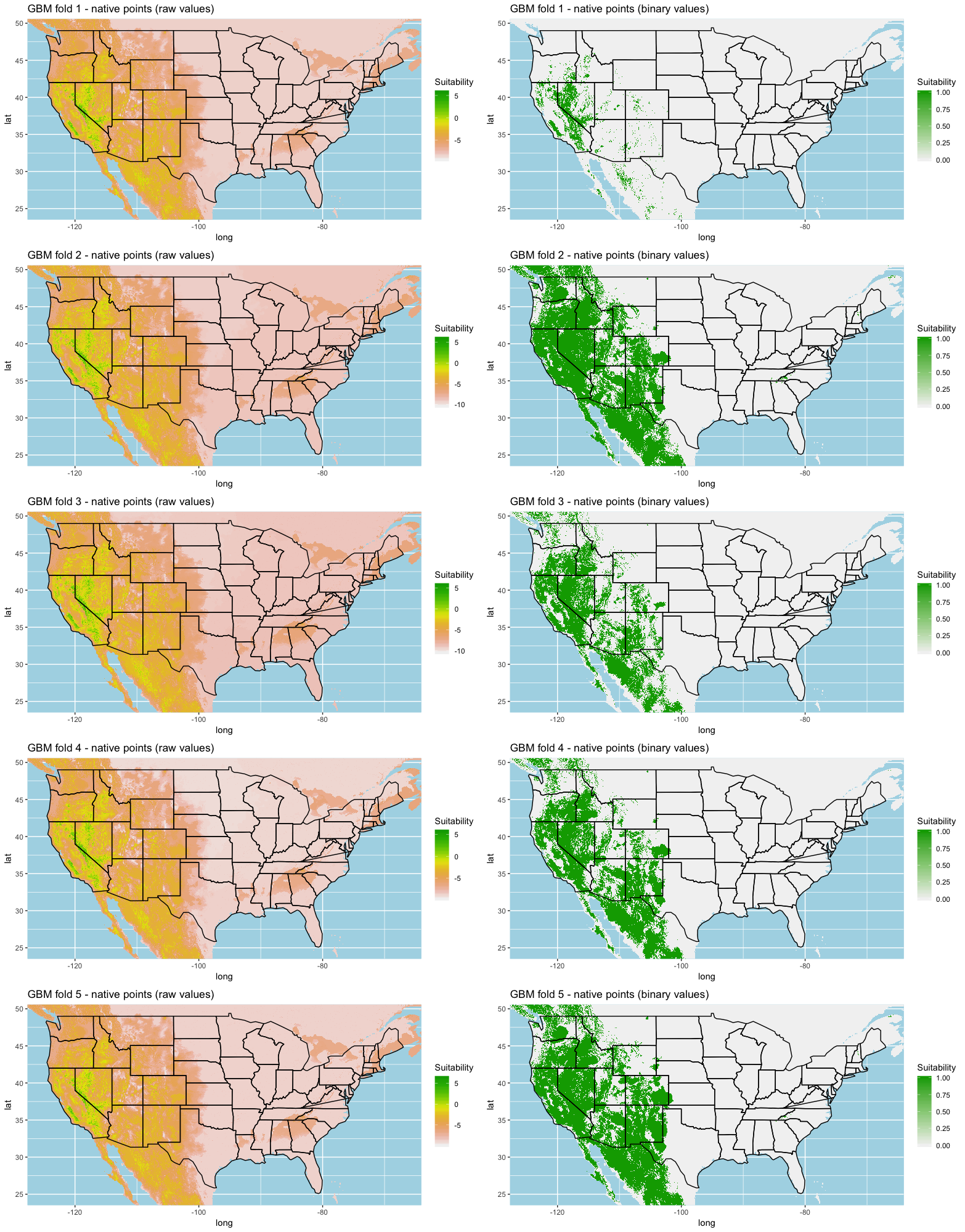

Supplement: Supplemental Information 12 [file peerj-09-11280-s012.png]

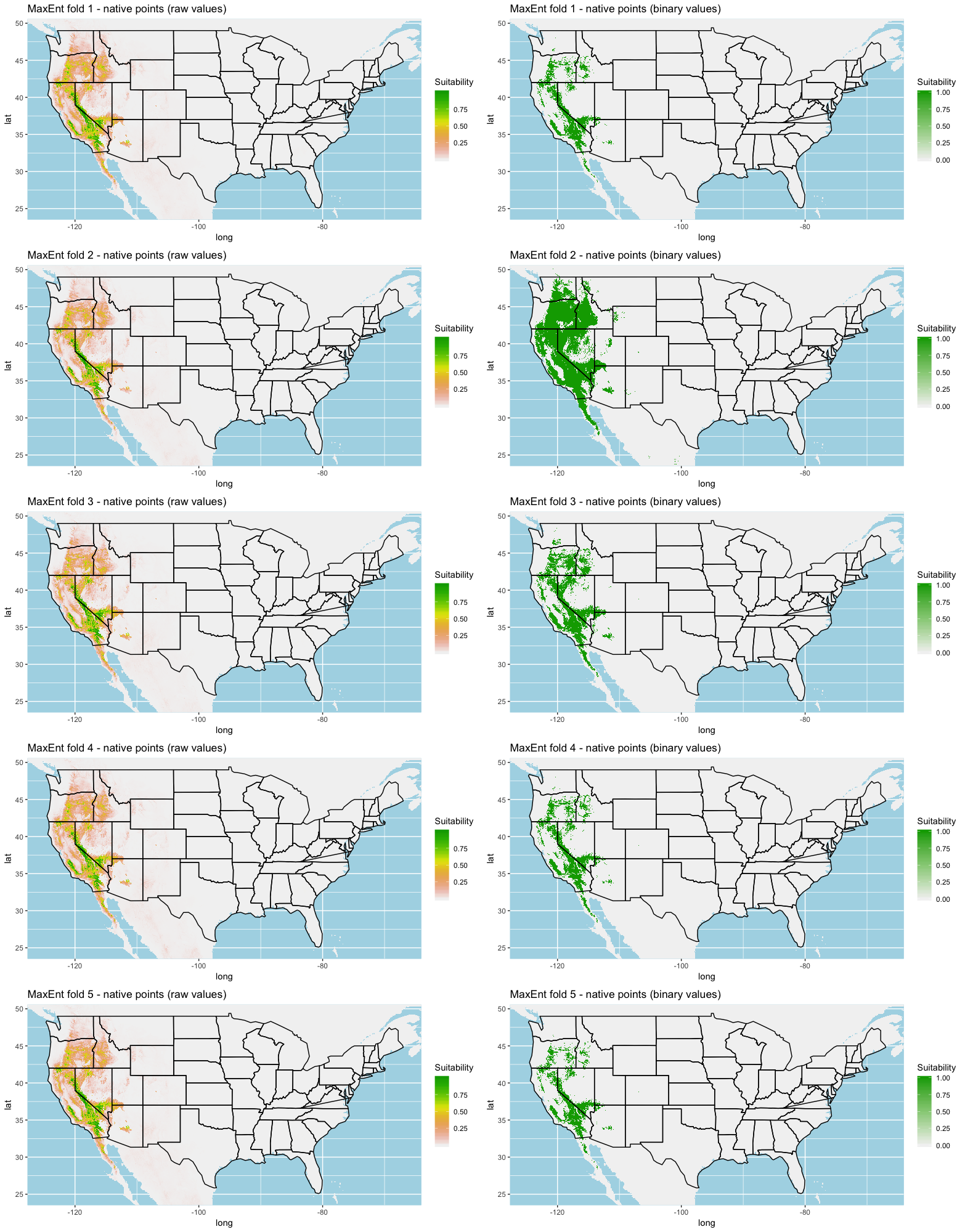

Supplement: Supplemental Information 13 [file peerj-09-11280-s013.png]

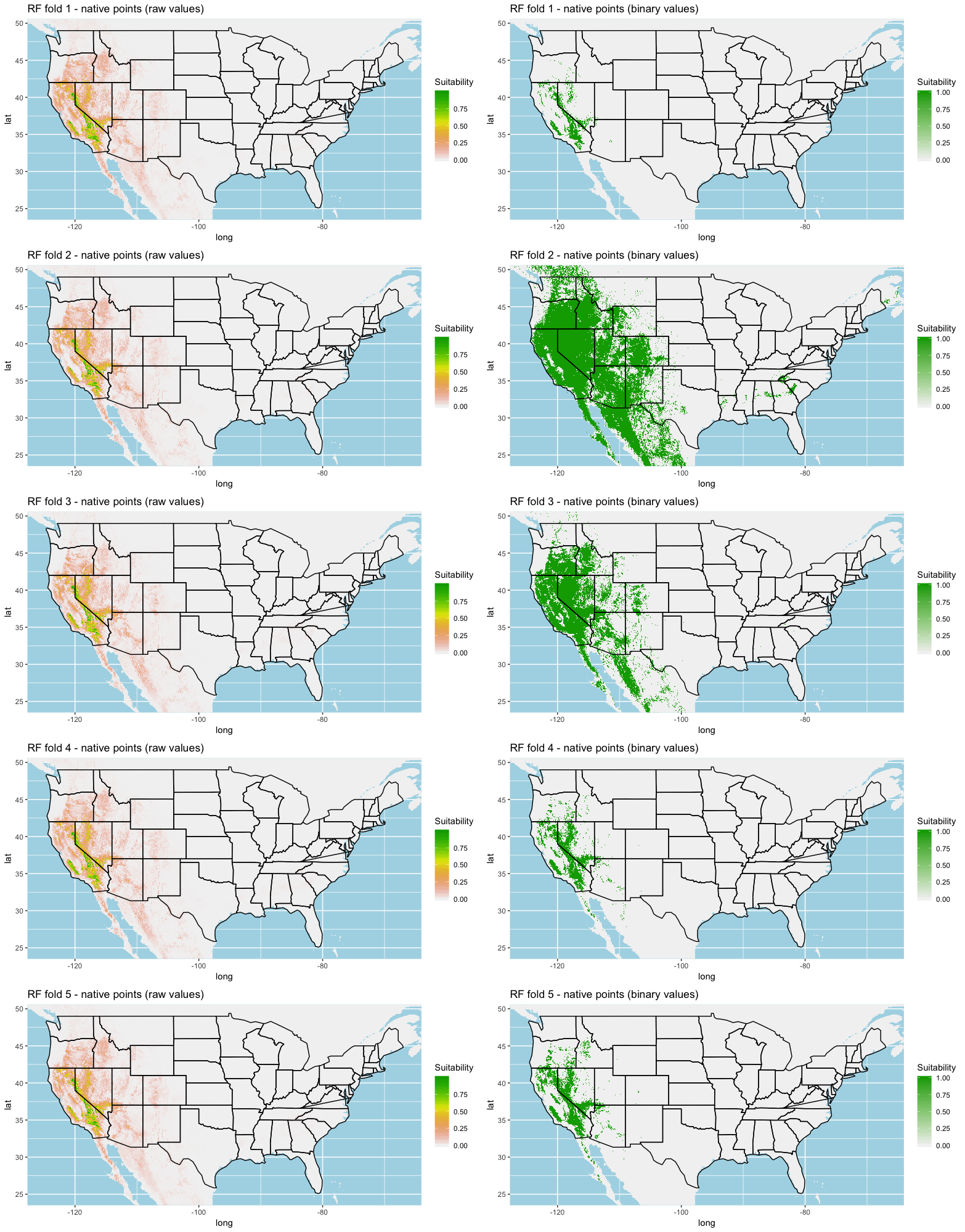

Supplement: Supplemental Information 14 [file peerj-09-11280-s014.png]

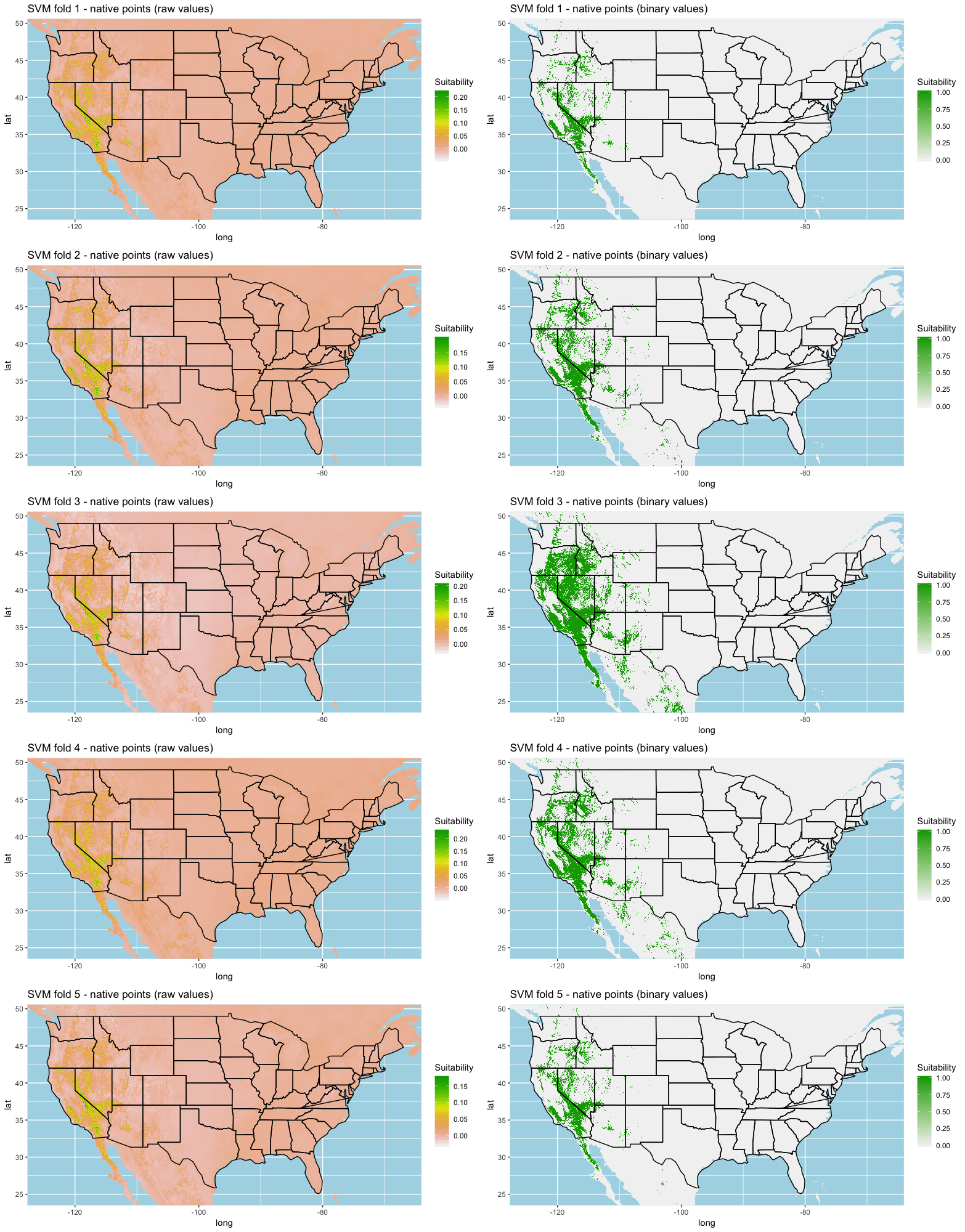

Supplement: Supplemental Information 15 [file peerj-09-11280-s015.png]

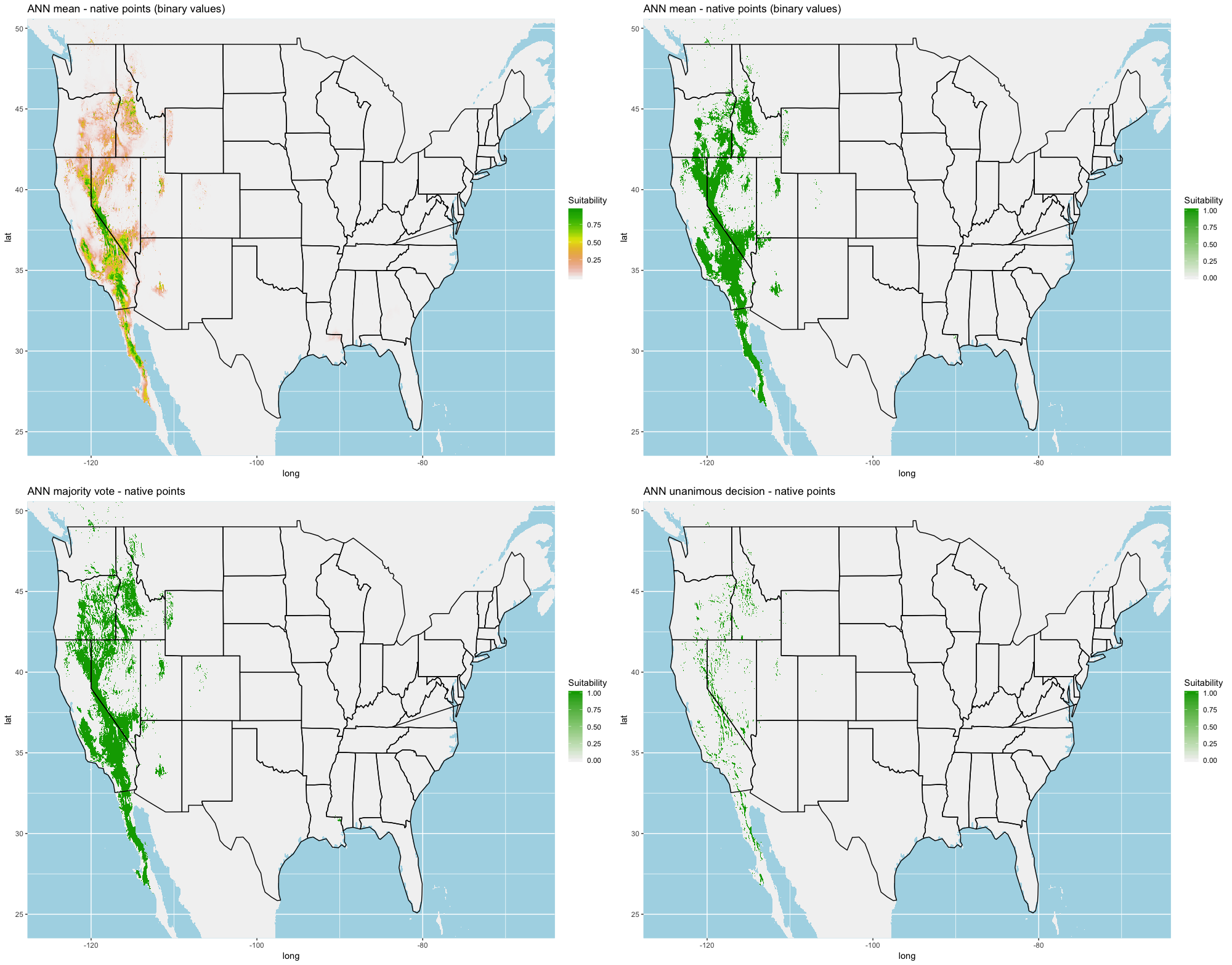

Supplement: Supplemental Information 16 [file peerj-09-11280-s016.png]

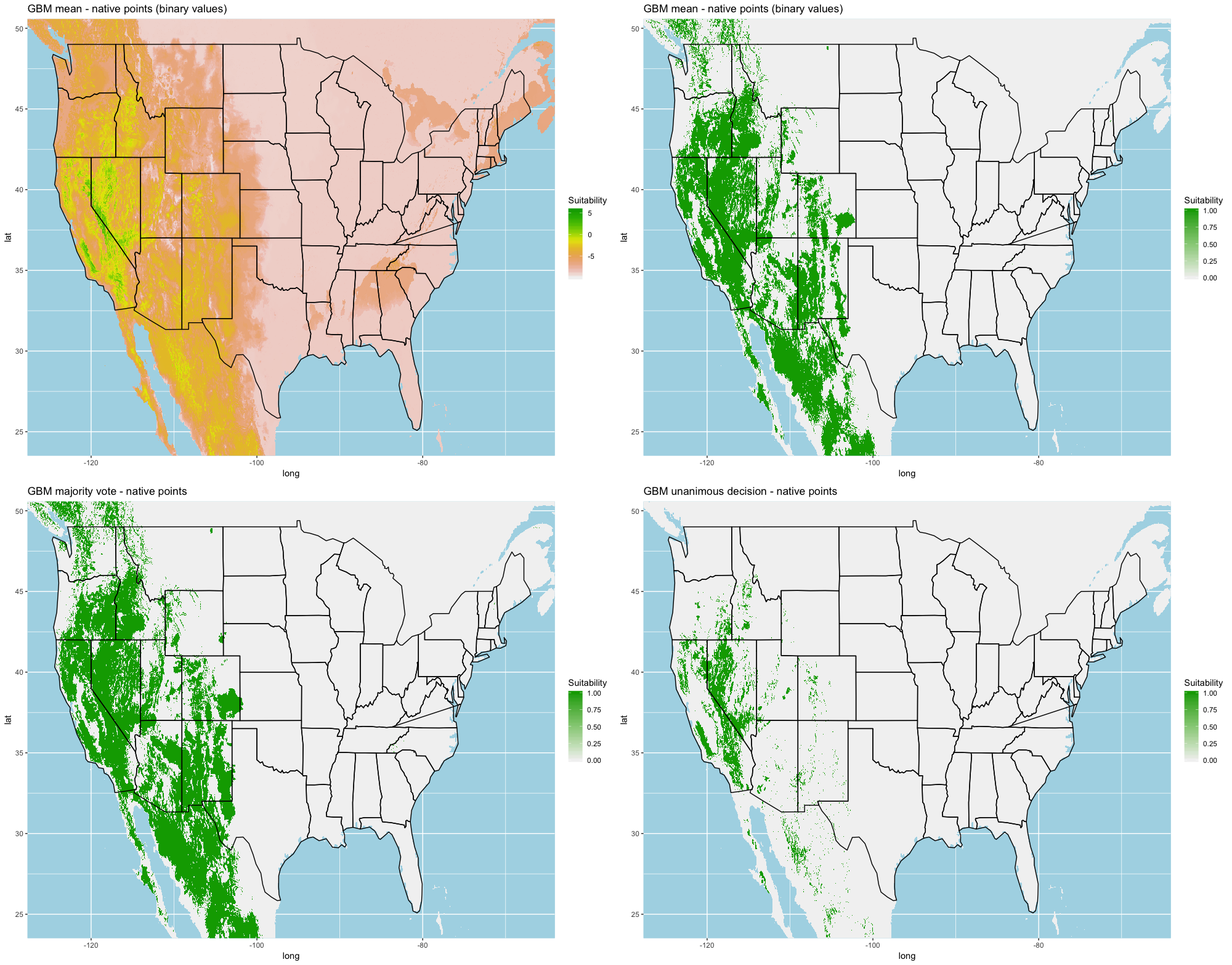

Supplement: Supplemental Information 17 [file peerj-09-11280-s017.png]

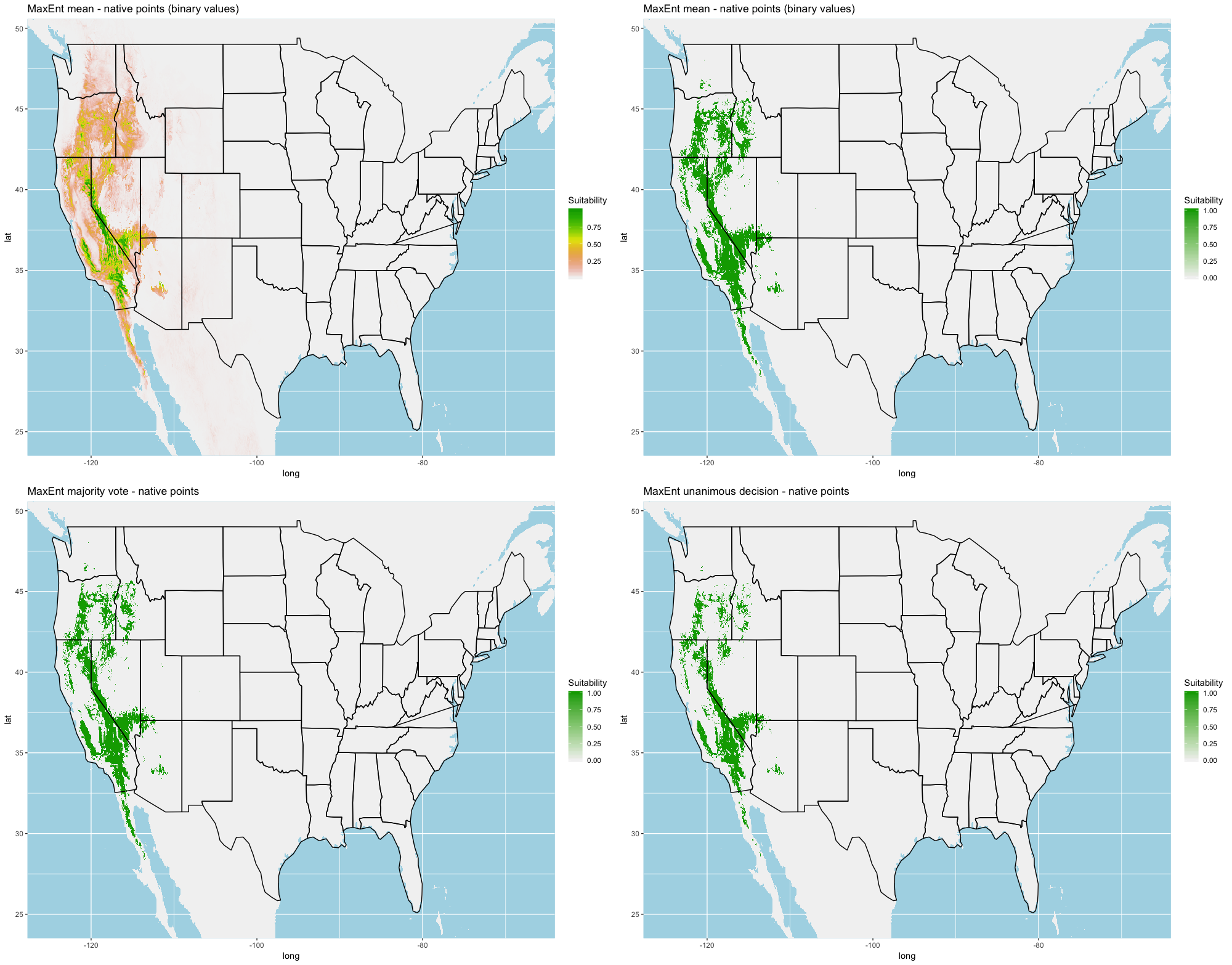

Supplement: Supplemental Information 18 [file peerj-09-11280-s018.png]

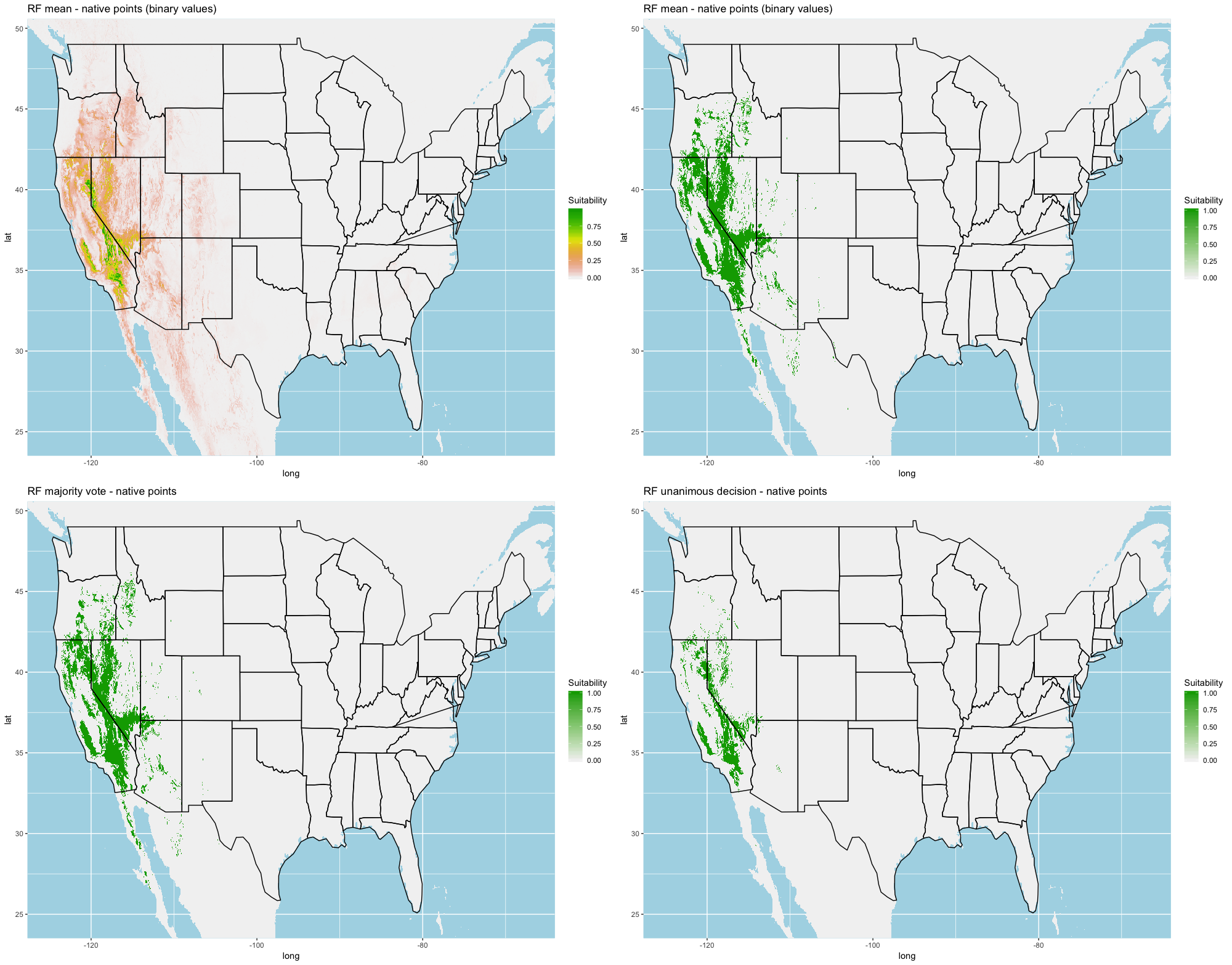

Supplement: Supplemental Information 19 [file peerj-09-11280-s019.png]

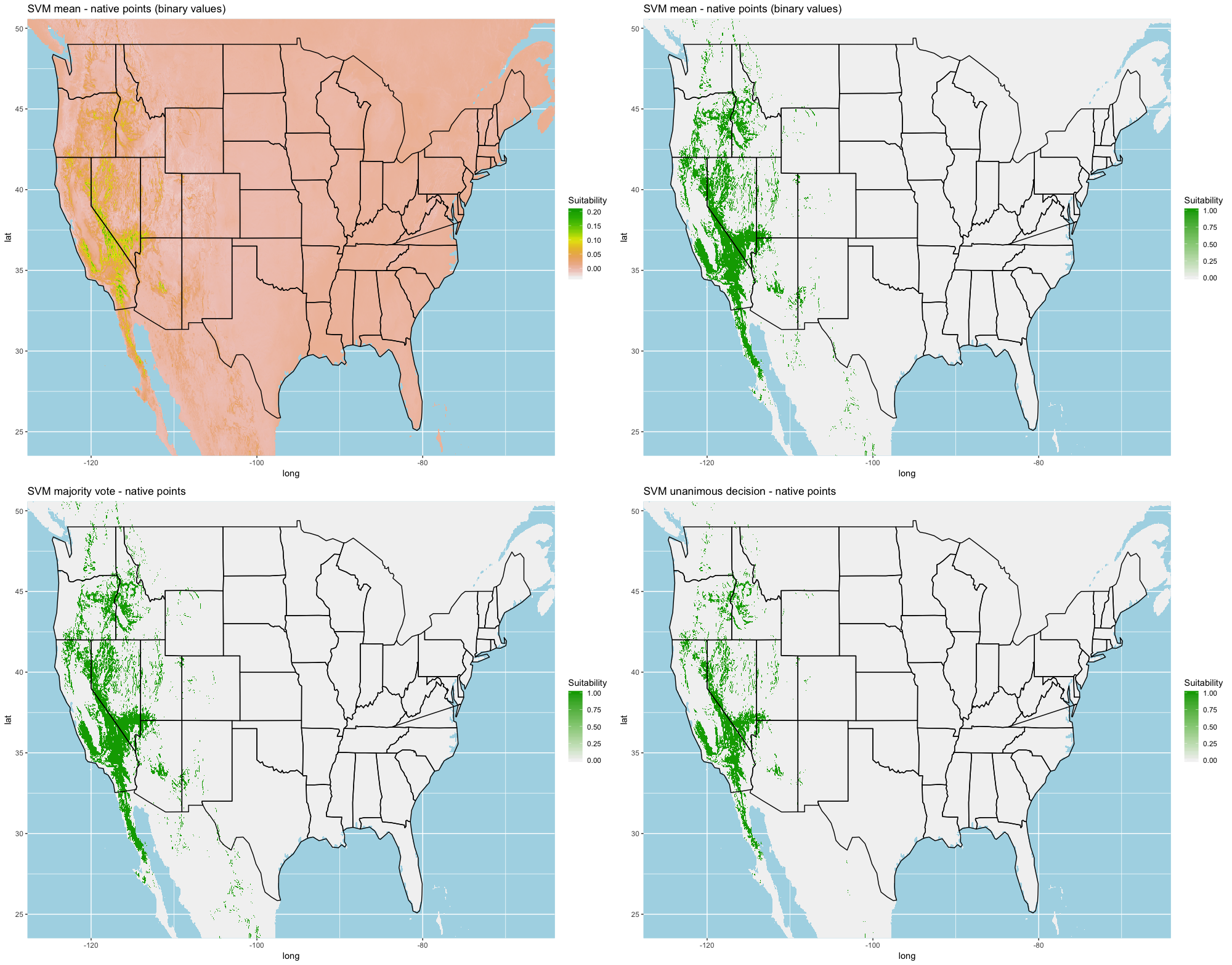

Supplement: Supplemental Information 20 [file peerj-09-11280-s020.png]
